# Blended peer-led research curriculum with AI integration improves postgraduate students’ academic performance and satisfaction: a quasi-experimental mixed-methods study

**DOI:** 10.1186/s12909-026-08576-2

**Published:** 2026-01-19

**Authors:** Zuhair S. Natto

**Affiliations:** Department of Dental Public Health, Faculty of Dentistry, King Abdulaziz University, Jeddah, Saudi Arabia

**Keywords:** Research methods education, Blended learning, Peer-assisted learning, Artificial intelligence, Postgraduate dental education, Mixed-methods

## Abstract

**Background:**

Research methods courses in health professions education are often perceived as abstract, didactic, and disconnected from practice, resulting in low engagement and limited skill transfer. Innovative pedagogies that integrate flipped learning, peer critique, and artificial intelligence (AI) may enhance learning but remain underexplored in postgraduate dental education. This study evaluated the effectiveness of a blended, peer-led, AI-integrated research methods curriculum compared with a traditional lecture-based approach.

**Methods:**

A quasi-experimental mixed-methods study was conducted during the Winter–Summer 2025 semester at the Faculty of Dentistry, King Abdulaziz University. First-year postgraduate dental students enrolled in a research methods course were assigned to either an intervention group (*n* = 26) that received a blended curriculum incorporating flipped video lectures, gamified peer critiques, Critical Appraisal Skills Programme (CASP)-based appraisals, faculty-led discussions, AI-assisted feedback, and proposal development, or a control group (*n* = 26) receiving traditional lectures. Quantitative outcomes included academic performance, critique score, and student satisfaction (Likert survey). Qualitative data were collected through focus groups and reflective responses, analyzed thematically.

**Results:**

Intervention students reported significantly higher overall satisfaction than the control group (Mean ± SD: 4.35 ± 1.56 vs. 3.35 ± 1.89; *p* = 0.043; 95% CI: 0.06 to 1.94). Differences in critique scores (9.23 ± 1.12 vs. 8.77 ± 0.96) and total academic performance (92.21 ± 18.50 vs. 85.50 ± 22.30) favored the intervention group but did not reach statistical significance. Satisfaction was moderately correlated with academic performance (around *r* ~ 0.6 in both groups, *p* < 0.001). Qualitative findings provided explanatory insight into these patterns, highlighting perceived clarity of course organization, active and collaborative learning, and improved preparedness for thesis work, alongside challenges related to workload intensity and course logistics.

**Conclusions:**

A blended, peer-led, AI-integrated research methods curriculum was associated with higher student satisfaction and more positive learning experiences compared with traditional lectures, while performance differences were modest. Mixed-methods integration revealed how engagement, structure, and perceived relevance shaped learning beyond what quantitative measures alone captured. Although resource-intensive, this model offers a promising framework for enhancing postgraduate research methods education. Further multi-institutional and longitudinal studies are needed to examine sustainability and generalizability.

**Supplementary Information:**

The online version contains supplementary material available at 10.1186/s12909-026-08576-2.

## Background

In the current era of evidence-based health care, the ability to critically appraise scientific literature and design sound research is a fundamental skill for professionals. Yet in many postgraduate health science programs, research methods education remains largely didactic and assessment-driven—dominated by lectures and summative exams. Several studies in health professions education have reported that traditional lecture-dominated approaches are associated with lower student engagement, reduced opportunities for inquiry, and limited transfer of theoretical knowledge to applied research skills, particularly in postgraduate settings [1–3]. Empirical studies further indicate that research methods courses in dental and health professions education are commonly experienced as conceptually demanding and abstract, while qualitative and survey-based evidence suggests that limited perceived relevance to clinical practice may reduce student motivation and promote surface learning approaches [3–5].

To address this challenge, it is essential to leverage innovative pedagogies and emerging technologies that make learning more interactive, relevant, and student-centered. Recent advances in educational technology—and especially artificial intelligence (AI)—offer new opportunities to enhance research training. Models such as flipped classrooms, peer feedback systems, and AI-assisted critique platforms can support more personalized, interactive, and student-driven learning [6, 7]. When thoughtfully integrated, these innovations have the potential to foster deep learning, ethical scholarship, and independent research capability [8, 9].

Despite a global shift toward learner-centered education, many postgraduate health programs still lack structured opportunities for autonomy, collaboration, and application of knowledge in real-world contexts [10]. Although recent scoping reviews and systematic reviews describe a rapidly expanding body of work on AI in dental and health professions education, applications remain unevenly distributed and are often descriptive or proof-of-concept. Relatively few rigorously evaluated interventions have focused specifically on research methods training or on curricula that deliberately integrate peer learning, AI-assisted feedback, and blended delivery in a theory-informed way.

Learning Management System (LMS) increasing interest in AI-enhanced learning, several important gaps remain unaddressed. First, there is limited empirical evidence on the efficacy of AI-generated feedback in supporting students’ development of critical appraisal skills in health professions education. Most existing studies describe feasibility or perceptions rather than measurable learning outcomes. Second, few investigations have examined how AI feedback interacts with other instructional components, such as peer critique, flipped learning, and structured appraisal tools. Finally, little is known about which elements of blended, inquiry-based research methods curricula contribute most strongly to student performance and engagement. The present study directly addresses these gaps by evaluating a blended, peer-led, AI-integrated research methods curriculum and analyzing performance, satisfaction, and student perceptions across components.

The study was guided by a conceptual framework integrating principles from adult learning theory and constructivist pedagogy. The framework posits that three core instructional components—peer learning, AI-assisted feedback, and blended delivery strategies (self-paced videos, in-class tasks)—interact to support students’ development of research literacy, critical appraisal competence, and learning engagement. Peer critique promotes active knowledge construction; AI feedback provides individualized, iterative refinement; and blended learning supports autonomy and flexibility. Together, these elements were hypothesized to produce superior learning outcomes compared to traditional lecture-based instruction.

In this study, a comprehensive blended curriculum for postgraduate students in health sciences is presented and evaluated. The curriculum integrates self-paced video learning, gamified peer critique, in-class study design tasks, structured Critical Appraisal Skills Programme (CASP) which can be downloaded through https://casp-uk.net/casp-tools-checklists/ [11], and AI-enhanced feedback. CASP is a checklist used in health care to assess the validity and usefulness of research studies. Grounded in adult learning theory and constructivist pedagogy [12–14], the study investigates how this blended, inquiry-based model affects research literacy, critical appraisal skills, and student engagement. The findings aim to provide empirical insight into how and which components are most effective in strengthening research methods education, particularly through the integration of AI tools.

This study makes several novel contributions to the literature on research methods education in the health professions. First, it evaluates a fully integrated, theory-informed curriculum that combines blended learning, structured peer critique, and regulated use of artificial intelligence within a postgraduate research methods course—elements that have largely been studied in isolation. Second, unlike prior work that focuses primarily on feasibility or student perceptions, this study employs a quasi-experimental mixed-methods design to examine both objective academic performance and student satisfaction. Third, the study explores how AI-assisted feedback functions alongside peer learning and established appraisal frameworks (e.g., CASP), providing empirical insight into the pedagogical role of AI beyond proof-of-concept demonstrations. Collectively, these contributions advance understanding of how AI can be meaningfully and responsibly integrated into research education to support deep learning and critical appraisal competence.

## Methods

### Study design

This study used a quasi-experimental, non-randomized convergent parallel mixed-methods design, chosen to allow quantitative assessment of academic performance, critique score, and student satisfaction alongside qualitative exploration of students’ learning experiences and perceptions. The study design and reporting follow recommended mixed-methods guidelines, ensuring that qualitative and quantitative components are integrated at interpretation and described transparently (Good Reporting of a Mixed Methods Study, GRAMMS) [15]. The mixed-methods approach enabled quantitative findings to be complemented and elaborated by qualitative insights, thereby enhancing the interpretive value of the results (GRAMMS Item 1, Supplementary File S1). Quantitative data were collected through standardized assessments and surveys, while qualitative data were obtained via focus group discussions and reflective responses. Data collection for both strands occurred concurrently, with neither strand prioritized, consistent with a convergent design. Integration of quantitative and qualitative results occurred at the interpretation stage, where qualitative themes were compared with quantitative trends to explain how specific curriculum components influenced observed outcomes (GRAMMS Item 4, Supplementary File S1). Each method is described in detail below with respect to sampling, data collection, and analysis procedures. Limitations related to each methodological strand, including single-researcher qualitative analysis, as well as insights gained from mixed-methods integration, are addressed in the Discussion (GRAMMS Items 5–6, Supplementary File S5).

### Setting and participants

The study was conducted in the Winter-Summer 2025 semester at the Faculty of Dentistry, King Abdulaziz University. Participants were first-year postgraduate dental students enrolled in the Research Methods course (DPHE 806). The course was offered in two parallel sections, and students were allocated to these sections through the routine course registration process, independent of the study. No randomization was performed. One section was designated as the intervention group, while the other served as the control group. The intervention group comprised 26 students; the control group consisted of a comparable concurrent cohort matched by prerequisites, curricula, and assessment requirements. To support comparability between groups, baseline characteristics were reviewed. Students in both the intervention and control cohorts had no formal research training prior to the course, aside from an internship research project required of all graduates. The two cohorts were drawn from the same program, completed identical prerequisites, and followed the same curriculum structure. Based on these criteria, the groups were considered educationally equivalent at baseline. Inclusion criteria included enrollment in the course and prior exposure to article critique using the Critical Appraisal Skills Programme (CASP) checklist. Students who did not consent or declined to participate in the qualitative component (focus groups/reflective responses) were excluded from those parts of the study. Ethical approval was obtained from the university’s Institutional Review Board (Approval No. 104-06−25).

### Educational intervention for experimental group

The experimental curriculum was a blended, student-centered research methods program integrating flipped learning, structured peer critique, active application tasks, and regulated use of AI tools. The intervention curriculum was structured across eight progressive components (Table 1). Core instructional strategies included:

**Table 1:** Summary of components in intervention vs. Control groups (Flipped Learning, peer Critique, AI use, etc.)

Phase/Component	Intervention (Blended, Peer-Led, AI-Integrated)	Control (Traditional, Lecture-Based, No AI/Peer)
1. Flipped & Flexible Learning	> 50 self-produced YouTube lectures aligned to LMS; students required to watch before class.	Weekly *live lectures* covering identical topics; YouTube videos optional for review, not required.
2. Gamified Group-Based Critique	Weekly group critiques of assigned articles; peer feedback emphasized; AI tools allowed; gamification with scores, dashboards, competition.	Weekly individual written critiques of the same articles; submitted to instructor; AI use prohibited; no gamification. However, there is peer feedback.
3. In-Class Application	Group study design challenges; one student presents per group; peer + instructor critique.	Instructor-led worked examples; brief individual exercises; instructor provides feedback only.
4. CASP Peer Appraisal Rounds	Students complete CASP appraisal on article of choice; exchange critiques with peers for structured discussion; AI use allowed.	Students complete CASP appraisal individually; submit to instructor; receive model answer or instructor summary; AI use prohibited.
5. Faculty-Led Discussion Rounds	Six rotating roundtable sessions; students present CASP appraisals; receive expert critique.	Six faculty lectures + Q&A; expert provides commentary
6. Online Expert Lectures	Live virtual sessions by external experts (AI in research, grant writing, stats, mixed methods); interactive discussion.	Identical sessions attended live; Q&A moderated by instructor; no small-group discussion.
7. Supplementary Skill Activities	Encouraged to submit proposals for real-world funding; complete take-home exam; obtain ethics certificate; supported by peer + AI coaching.	Encouraged to submit proposals for the blackboard. instructor-provided handouts; no peer or AI coaching.
8. Final Proposal & Oral Defense	Comprehensive research proposal + oral defense before panel; students supported by prior peer critique & AI feedback.	Same proposal + oral defense before panel; no AI rehearsal and no structured peer critique in preparation.
Overall Learning Model	Blended, inquiry-based, constructivist; peer-led, gamified, AI-enhanced.	Lecture-centered, instructor-led, traditional; no peer critique, no gamification, no AI.
AI Role	Allowed in critiques, peer appraisal, proposal drafting support.	Prohibited in all graded components (honor code enforced).
Feedback Sources	Multi-source: peer, AI, dashboards, faculty.	Instructor-only (generalized feedback, exemplar solutions).
Engagement Strategy	Gamification, rotating group roles, interactive tasks.	Attendance + passive lecture; optional Q&A.
Assessment	Same rubrics for MCQs, critiques, proposals, oral defense.	Same rubrics for MCQs, critiques, proposals, oral defense.

- Pre-class learning: Self-paced video lectures on foundational research concepts (e.g., study design, hypothesis formulation, critical appraisal) [16].
- Peer-led critique activities: Weekly small-group article appraisals using the Critical Appraisal Skills Programme (CASP) framework, supported by structured peer feedback processes and gamification using a standardized scoring rubric (Supplementary File S2) to enhance engagement. AI support (ChatGPT) was permitted to improve clarity and organization of written critiques without replacing student reasoning.
- In-class active tasks: Group design exercises with peer and instructor feedback, plus faculty-facilitated discussion rounds on study designs to deepen analytical thinking.
- Expert sessions and supplementary activities: Live expert lectures on relevant topics (e.g., AI in research, statistical decision-making), take-home design assessments, research proposal development, and research ethics certification.
- Final assessment: Individual development and oral defense of a comprehensive research proposal with iterative refinement incorporating feedback from peers, AI tools, and instructors.

The intervention was standardized across all components with predefined learning objectives, timelines, and assessment criteria. Detailed curricular materials and implementation guides are provided in (Supplementary File S3).

Across the intervention components, reflection and peer feedback were intentionally embedded as core instructional strategies grounded in constructionist learning theory. Reflection prompts and iterative critique tasks required students to externalize their reasoning, evaluate methodological decisions, and progressively refine their understanding through engagement with authentic research problems. Peer feedback—particularly during group-based critiques and CASP appraisal rounds—was designed to facilitate collaborative knowledge construction, expose students to diverse analytic perspectives, and support learning through social interaction and dialogue, rather than serving solely as an assessment mechanism. The control group followed a traditional lecture-based curriculum covering the same content domains, but without peer-led components, gamification, or AI integration (Table 1).

### Assessment instruments and data collection

#### Quantitative Measures

- Critique Score: Students submitted either manual critique or AI-assisted critiques of selected articles. A standardized rubric assessed clarity, evidence-based reasoning, depth of analysis, and adherence to CASP criteria. Grading was done by two independent raters blinded to group allocation; inter-rater reliability was calculated using Cohen’s kappa (κ), which was above 0.70, indicating substantial agreement.
- Student Satisfaction: An investigator created post-course Likert-scale survey (5-point Likert) measured satisfaction with the learning experience, perceived learning gains, and usability of the AI tool (Supplementary File S4). Prior to deployment, the survey was pilot-tested with five postgraduate dental students who took the course before and not enrolled in the study cohort for face validity, two educational experts for content validity. Minor wording modifications were made based on their feedback to improve clarity.
- Academic Performance: Final course grades and critique scores from both intervention and control groups were collected, using identical rubrics and grading standards (Supplementary File S5 & S6). All components were equally weighted except for the final proposal & oral defense. Student assessments were conducted by multiple faculty members, including the course instructor. All faculty graders used identical rubrics and scoring guidelines to ensure consistency. Students were informed during course orientation that the instructor was part of the research team, and they were assured that their decision to participate—or not participate—in the study would not influence their grades. All grading was completed prior to any analysis, and the research dataset was anonymized to prevent bias.ru.

#### Qualitative Measures

Focus Group Discussions: Two focus group discussions were conducted—one with students from the intervention group and one with students from the control group—to ensure representation across both pedagogical models. Each focus group included 6–8 participants, recruited voluntarily after all course assignments and grading were completed to avoid any influence on academic evaluation. Sessions lasted approximately 60 min and were conducted within one week of course completion.

Focus groups were facilitated by the course director. A semi-structured interview guide was used, with prompts exploring students’ experiences with peer critique (e.g., “Describe how you engaged with peer feedback and how it influenced your learning”), perceptions of AI tool usability and value (e.g., “How helpful was AI feedback in improving your critique?”), reflections on course components, and recommendations for improvement. The same set of core questions was used across all groups to ensure comparability, with minor follow-up probes adapting organically to each discussion.

All sessions were audio-recorded, transcribed verbatim, and anonymized prior to analysis. Member checking was conducted by sharing a summary of preliminary themes with a subset of participants to confirm accuracy and credibility of interpretation.

### Data analysis

- *Sample size*: This study was conducted as a pilot quasi-experimental investigation, and therefore no a priori power analysis was performed. The sample size of 26 students per group reflected the total number of students enrolled in each cohort during the academic year. Pilot studies commonly prioritize feasibility and preliminary effect estimation rather than hypothesis testing; thus, the current sample size is appropriate for generating initial estimates of effect sizes and informing future adequately powered studies.
- *Quantitative Analysis*: Descriptive statistics (means, standard deviations, score ranges) for all outcome variables. Independent t-test (or Mann-Whitney U test if data don’t meet assumptions) used to compare intervention vs. control groups on critique scores, satisfaction, and academic performance. Correlation analyses (Pearson’s r) to examine relationships between satisfaction and the critique scores/final course grades. Quantitative analyses were performed using SPSS (IBM Corp., Armonk, NY, USA), with significance level set at *P* < 0.05.
- *Qualitative Analysis*: Thematic analysis following Braun & Clarke’s (2006) six-phase framework: (1) familiarization with data, (2) generating initial codes, (3) searching for themes, (4) reviewing themes, (5) defining and naming themes, (6) producing the report. Coding was iterative. Reflexivity memos were kept. One researcher coded all transcripts using an iterative thematic analysis approach. An initial set of open codes was generated, followed by development of a shared codebook. The refined codebook was then applied to all transcripts. Data saturation was reached when no new themes emerged in the final focus group. To enhance credibility, a subset of participants reviewed a summary of themes (member checking), and coder triangulation was employed throughout analysis. The final codebook is provided in Supplementary File S7.
- *Integration of Mixed Methods Findings*: For this convergent mixed-methods study, qualitative and quantitative findings were integrated during the interpretation phase. Quantitative results (e.g., academic performance measures, satisfaction scores) were compared and contrasted with themes emerging from the focus group data to identify areas of convergence and complementarity. Narrative integration was achieved by aligning qualitative insights with quantitative patterns and explaining how participants’ experiences and perceptions helped contextualize and interpret measured outcomes.

### Ethical considerations

This study was approved by the research ethical committee at King Abdulaziz University (#104-06−25). Participation was voluntary and informed consent was obtained from all students (Supplementary File S8). Data collected (survey responses, reflections, critiques, transcripts) were anonymized.

## Results

### Quantitative results

#### Overall satisfaction and academic performance

Critique performance did not differ significantly between the intervention group (M = 9.23, SD = 1.12) and the control group (M = 8.77, SD = 0.96), t(48.86) = 1.59, *p* = 0.12, 95% CI [− 0.12, 1.04] (Table 2). Total academic performance was higher in the intervention group (M = 92.21, SD = 18.50) than the control group (M = 85.50, SD = 22.30), but the difference was not statistically significant, t(48.35) = 1.18, *p* = 0.24, 95% CI [− 4.71, 18.13]. Overall satisfaction was significantly higher in the intervention group (M = 4.35, SD = 1.56) than the control group (M = 3.35, SD = 1.89), t(48.27) = 2.08, *p* = 0.043, 95% CI [0.06 to 1.94].

**Table 2:** Overall student satisfaction and total performance scores by group (intervention vs. control)

Outcome	Intervention (Mean ± SD)	Control (Mean ± SD)	t(df)	*p*-value	95% CI (LL–UL)
Overall Satisfaction	4.35 ± 1.56	3.35 ± 1.89	2.08 (48.27)	0.043*	0.06 to 1.94
Critique Scores	9.23 ± 1.12	8.77 ± 0.96	1.59 (48.86)	0.12	–0.11 to 1.03
Total Performance Score	92.21 ± 18.50	85.50 ± 22.30	1.18 (48.35)	0.24	–4.43 to 17.85

*Significant at *p* < 0.05

#### Course component ratings

**Fig. 1:**
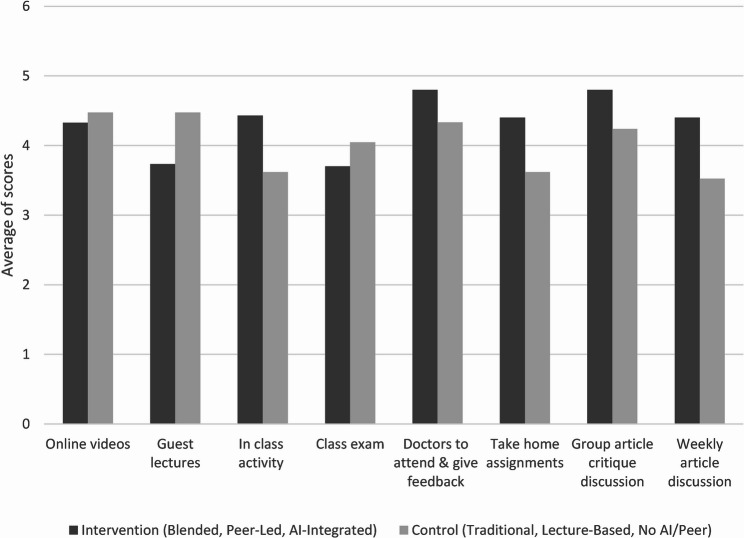
Comparison of student ratings for course components between intervention and control groups

Mean ratings for individual course components differed descriptively between groups (Fig. 1). Intervention students rated interactive and feedback-oriented components more favorably, including in-class activities, faculty feedback, take-home assignments, and group critique discussions. In contrast, control students rated traditional components such as online videos, guest lectures, and examinations slightly higher. These differences are presented descriptively and were not subjected to inferential testing.

Qualitative findings help contextualize these patterns, with intervention students emphasizing the value of active engagement and iterative feedback, while control students expressed greater familiarity with traditional instructional formats but lower engagement.

#### Course Objectives, Materials, and knowledge gain

**Fig. 2:**
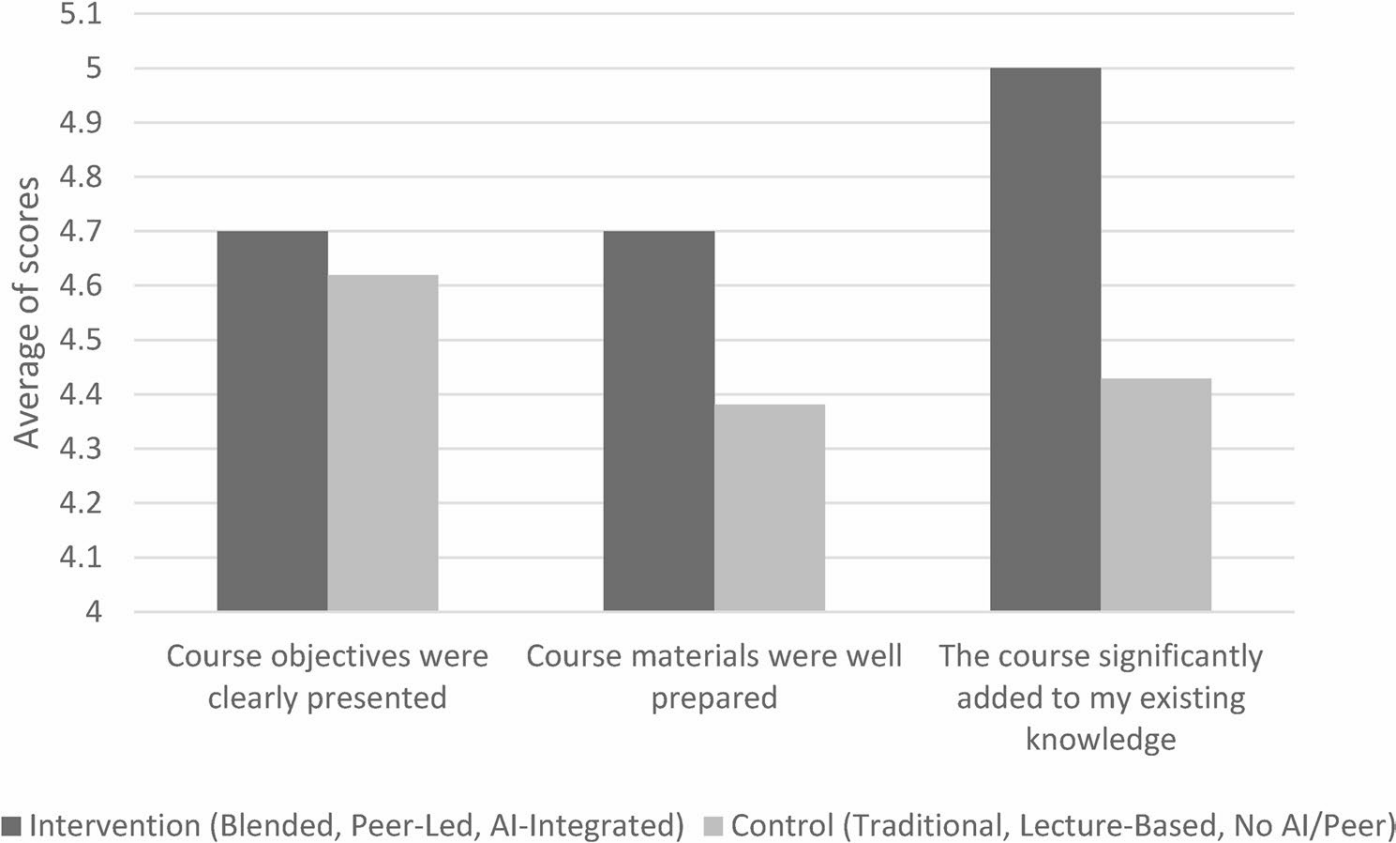
Comparison of course objectives, materials, and knowledge gain between intervention and control groups

Descriptively, the intervention group reported higher perceived clarity of course objectives, preparedness of course materials, and self-reported knowledge gain compared with the control group (Fig. 2). These findings reflect students’ perceptions of the learning experience rather than objective measures of knowledge acquisition. Qualitative themes related to course organization and clarity provide explanatory context for these perceptions.

#### Gender differences

When stratified by gender (Table 3), female students reported slightly higher satisfaction scores than male students in both groups; however, these differences were not statistically significant in either the intervention or control cohorts.

**Table 3:** Student overall satisfaction by gender within intervention and control groups

Group	Male (Mean ± SD, *n* = 12)	Female (Mean ± SD, *n* = 14)	t(df)	*p*-value	95% CI (LL–UL)
Intervention	4.10 ± 1.70	4.57 ± 1.40	0.80 (23.20)	0.12	–0.39 to 1.33
Control	3.00 ± 2.10	3.64 ± 1.65	0.84 (21.62)	0.08	–0.46 to 1.82

*CI* = confidence interval for the difference in means (female – male)

No comparisons reached statistical significance

#### Correlation between satisfaction and performance

Pearson’s correlation analysis showed a moderate positive relationship between satisfaction and academic performance in both groups (intervention: *r* = 0.65, *P* < 0.001; control: *r* = 0.60, *P* = 0.001). A similar pattern was observed for critique scores (intervention: *r* = 0.60, *P* < 0.001; control: *r* = 0.45, *P* = 0.02) (Table 4). Qualitative findings suggest that this relationship may be mediated by engagement and perceived relevance, as students who described greater involvement in learning activities also reported higher confidence and satisfaction.

**Table 4:** Correlation between satisfaction and academic performance within each study group

Group	Satisfaction vs. Total Performance (*r*, *p*-value)	Satisfaction vs. Critique Score (*r*, *p*-value)
Intervention	*r* = 0.65, *p* < 0.001*	*r* = 0.60, *p* = 0.001*
Control	*r* = 0.60, *p* = 0.001*	*r* = 0.45, *p* = 0.02*

*Significant at *p* < 0.05.

#### Final course grade distribution

To confirm that the grading system remained equitable and that participation in qualitative components did not influence academic grading, final course grade distributions were compared between the two groups using identical rubrics and scoring criteria (Supplementary File S5 & S6). As shown in Table 5, the intervention group had a higher proportion of students achieving an A grade, consistent with their higher overall performance scores. Comparison of final course grade distributions using identical rubrics demonstrated comparable grading patterns across groups, indicating that participation in qualitative or peer-led activities did not influence grading practices. This supports the internal validity of performance comparisons.

**Table 5:** Comparison of final course grades between intervention and control groups

Grade Category	Intervention (*n* = 26)	Control (*n* = 26)
A (≥ 90%)	14 (53.8%)	10 (38.5%)
B (80–89%)	8 (30.8%)	9 (34.6%)
C (70–79%)	4 (15.4%)	7 (26.9%)
D/F (< 70%)	0	0

### Qualitative results

To complement the quantitative findings, thematic analysis of focus group discussions and written reflections was conducted separately for the intervention and control groups. Participant identifiers (e.g., INT-P05 = Intervention participant 5; CON-P12 = Control participant 12) are used to ensure transparency and to demonstrate grounding of themes in the data. Qualitative findings are presented to complement and extend the quantitative results by exploring how students experienced the course structure, learning activities, and assessment demands. Thematic analysis focused on identifying mechanisms underlying observed satisfaction and engagement patterns, as well as areas not fully captured by quantitative measures.

### Intervention Group Perceptions

1. Course Organization and Clarity.

Intervention students consistently described the course as clearly structured, with transparent expectations and logical sequencing. Consistent with higher satisfaction ratings observed quantitatively, intervention students consistently described the course as clearly structure.

*“The course was very organized and clear from the beginning; everything was well detailed and comprehensive.”* (INT-P03).

2. Instructor Effectiveness and Support.

Faculty were perceived as supportive and effective at simplifying complex concepts, which helped reduce anxiety about research methods.

*“Prof X’s teaching style is excellent; the course wouldn’t be as enriching without his presence.”* (INT-P11).

3. Active and Diverse Learning Methods.

Students valued the blended, interactive components—peer critique, YouTube videos, AI guidance, and in-class study design tasks. Consistent with higher satisfaction ratings observed quantitatively, students valued the blended and interactive component.

*“I liked the variety of exercises—each one taught me something new and made me think in a different way.”* (INT-P07).

4. Practical Application and Research Preparedness.

The iterative critique and design tasks helped students feel better prepared to engage in real research and thesis development.

*“It gave me a clue where to start planning my thesis and how to choose a topic.”* (INT-P14).

5. Collaborative Learning Environment.

Students emphasized the benefits of peer collaboration, describing group work as supportive and enriching.

*“The cooperation between students as we worked in groups was one of the most beneficial aspects.”* (INT-P02).

### Control Group Perceptions

Control group themes of workload burden, logistical challenges, and limited engagement provide explanatory context for lower satisfaction ratings observed quantitatively. These findings suggest that dissatisfaction was driven less by content difficulty and more by course delivery and perceived misalignment with learner needs.

1. Perceived Excessive Workload.

Control students commonly reported feeling overwhelmed by the number and pacing of critiques and assignments. These perceptions align with lower satisfaction ratings in the control group and provide explanatory context for the quantitative findings.

*“The number of assignments was too much; after some weeks*,* I just wanted to submit without quality because of the load.”* (CON-P05).

2. Exam Structure and Timing.

Concerns were raised regarding exam timing and completion expectations.

*“We didn’t have enough time to solve the final exam; it should have been longer or designed differently.”* (CON-P09).

3. Guest Lectures and Online Session Engagement.

External speaker sessions were viewed as less engaging or difficult to follow.

*“The guest speaker sessions were boring and hard to understand; I learned more from our main instructors.”* (CON-P12).

4. Course Logistics and Delivery Issues.

Control students described challenges with LMS navigation, deadlines, and scheduling.

*“The Blackboard system was confusing.”* (CON-P03).

5. Mismatch With Learner Needs.

Some students felt that content began at a level too advanced for beginners or not aligned with their specialty.

*“Some sessions started at a level beyond beginners…”* (CON-P01).

## Discussion

This study demonstrates that, compared with a traditional lecture-based approach, a blended, peer-led, AI-integrated curriculum produced significantly higher student satisfaction, and more favorable perceptions of the course, with a modest (non-significant) trend toward higher academic performance among postgraduate dental students. The intervention group rated the clarity of objectives, quality of materials, active learning tasks, and perceived knowledge gain more positively, suggesting that this pedagogical design helps address common challenges in research methods instruction—namely abstraction, low engagement, and difficulty translating theory into practice.

### Peer-assisted learning and student engagement

The findings align with prior evidence suggesting that peer-assisted learning (PAL) is associated with improvements in learning outcomes across health professions education. A recent scoping review of systematic reviews reported that PAL—encompassing peer teaching, structured feedback, and group critique—has been consistently associated with enhanced academic performance, learner satisfaction, and skill development across a range of health professional programs [17]. These patterns are reflected in our study, where students in the intervention group described greater collaboration, clearer feedback processes, and deeper engagement with research appraisal tasks.

In addition, emerging literature on AI-enabled pedagogy highlights how AI tools can support digital literacy, personalized feedback, and learner autonomy in postgraduate education. Recent studies indicate that AI-supported critique, adaptive guidance, and digital competence development may further enhance learner engagement and confidence in research-oriented tasks [18–20]. These findings are consistent with this growing body of work, suggesting that integrating AI into peer-learning ecosystems may offer complementary benefits, particularly within blended, inquiry-based research methods curricula. Similarly, meta-analyses have found that PAL interventions are associated with modest but meaningful gains in examination performance relative to conventional methods [21]. The positive reception and performance advantages observed in our study likely reflect the active engagement, cognitive congruence, and scaffolded peer interaction inherent in PAL models [22].

### Role of artificial intelligence in dental education

The observed benefits of AI-enhanced critique and feedback are consistent with recent reviews highlighting AI’s role in dental and health professions education. Systematic reviews note that AI-powered tools can enhance diagnostic accuracy, provide individualized feedback, and support student learning, although institutional readiness and methodological heterogeneity remain challenges [23, 24]. Other work emphasizes that AI offers opportunities for automating assessment and tailoring educational content, but highlights the importance of addressing ethical, pedagogical, and faculty development issues [25]. In our study, qualitative findings echoed these caveats: while many students appreciated AI’s feedback and iterative support, some reported that workload, lecture alignment, or mismatch with prior knowledge limited their engagement. These results underscore the need to balance innovation with realistic workload expectations, faculty preparation, and clear ethical guidelines.

### Satisfaction–performance link and gender differences

There was a moderate positive correlation between student satisfaction and academic performance. Although this association does not imply causation, it aligns with educational engagement theory, which conceptualizes engagement as a multidimensional construct involving behavioral, emotional, and cognitive components [26]. According to this framework, emotionally positive experiences—such as satisfaction, perceived relevance, and enjoyment—can enhance students’ willingness to invest effort (behavioral engagement) and engage more deeply with academic content (cognitive engagement). These mechanisms help explain why students who report higher satisfaction often demonstrate stronger academic performance.

In this study, intervention-group students described greater clarity, collaborative learning, and meaningful feedback—features known to foster emotional and cognitive engagement. Such engagement-enhancing conditions may contribute to the observed association between satisfaction and performance, consistent with engagement theory and prior research in health professions education. While female students reported slightly higher satisfaction, differences were not statistically significant, suggesting broad acceptability across genders. Larger multi-institutional studies may help clarify potential subgroup effects.

### Strengths and limitations

Strengths of this study include its mixed-methods, quasi-experimental design, which enabled triangulation of quantitative and qualitative findings and allowed exploration of both outcomes and learning processes. The use of equivalent content coverage, assessment criteria, and grading rubrics across groups enhanced internal validity. Integrating multiple instructional strategies—including flipped learning, peer critique, and AI-assisted feedback—reflected an authentic educational environment rather than a single isolated intervention.

The findings should be interpreted within their institutional and cultural context. The study was conducted within a postgraduate dental program at a public university in Saudi Arabia, characterized by cohort-based learning, close faculty supervision, and strong expectations for engagement and professionalism. Elements of the hidden curriculum—such as norms surrounding participation, peer collaboration, and responsiveness to feedback—may have influenced student engagement and learning experiences. While these contextual factors may have supported the effectiveness of the intervention, they may also limit transferability. Future studies across diverse institutional and cultural settings are needed to examine contextual adaptation.

Several limitations should be acknowledged. The relatively small, single-institution sample constrains generalizability, and the non-randomized design means that selection bias cannot be excluded. Although groups were educationally equivalent at baseline, unmeasured differences may have influenced outcomes. Student feedback also indicated that the intervention was workload-intensive, and logistical challenges—including scheduling, learning management system usability, and coordination of external lectures—posed barriers to feasibility. Furthermore, implementation of a blended, peer-led, AI-integrated curriculum requires faculty training, ongoing support, and dedicated time for preparation and feedback, which may limit scalability in resource-constrained settings.

Measurement limitations should also be considered. While expert review provided qualitative evidence of content validity for the satisfaction survey, quantitative content validity indices (e.g., I-CVI or S-CVI) were not calculated. This may limit the psychometric rigor of the instrument and comparability with validated scales.

With respect to qualitative methods, only two focus group discussions were conducted; although thematic saturation was monitored and core patterns were identified, additional groups could have provided greater depth. The focus groups were facilitated by the course director, which may have introduced social desirability bias despite mitigation strategies such as neutral questioning, assurances of confidentiality, and reflexive analysis. This facilitation context may have contributed to more positive themes in the intervention group and more critical themes in the control group.

Finally, because students in both cohorts were aware of the parallel curriculum formats, perceived disadvantage among control-group students and informal communication between cohorts may have influenced subjective outcomes, particularly satisfaction ratings. The outcomes assessed were also short-term; longer-term impacts on thesis quality, publication output, or grant activity were not evaluated.

### Implications and future research

The findings suggest that content-heavy courses like research methods can be made more effective by adopting blended, peer-led, AI-augmented models. Success requires scaffolding support for learners, transparent guidelines around AI use, balanced workload, and adequate faculty training and infrastructure. AI tools in particular hold promise, but require oversight, ethical governance, and integration into existing pedagogical frameworks [23].

An important implication of these findings concerns the ethical integration of AI tools in postgraduate education. As AI-supported feedback becomes more common, educators must establish clear guidelines regarding appropriate use, academic integrity, data privacy, and the boundaries between human and machine-generated work. Ethical integration also requires transparency: students should understand when and how AI is being used, the limitations of AI-generated outputs, and the continued necessity of human judgment in research critique and decision-making. Faculty development is equally critical, as instructors must be equipped to guide students in responsible use, detect overreliance or misuse, and model reflective digital literacy. Embedding AI within a coherent pedagogical and ethical framework will be essential for ensuring that these tools enhance rather than replace critical thinking, uphold academic standards, and promote equitable learning experiences in future curriculum design.

Notably, the qualitative findings illuminated why the intervention was well-received – students valued the clarity, interactivity, and relevance – factors that are not captured by the exam scores alone. This integrative understanding, facilitated by our mixed-methods design, underscores the importance of gathering student feedback in addition to test metrics.

Future research should replicate and expand this study across multiple institutions and disciplines to strengthen generalizability. Factorial or additive designs could isolate the specific contributions of peer critique, AI feedback, gamification, and flipped learning. Longitudinal studies are also needed to determine whether gains in critical appraisal and research design are sustained and whether they lead to higher-level outcomes such as quality theses, publications, or funded grants.

## Conclusion

This study provides evidence that a blended, peer-led curriculum with AI integration was associated with higher satisfaction and performance compared with traditional lecture-based pedagogy. The findings suggest that incorporating flipped learning, peer critique, and AI-assisted feedback may help address persistent challenges in teaching research methods, making courses more engaging, practical, and effective. While such interventions demand greater resources, planning, and faculty development, the gains in student engagement, clarity, and learning effectiveness highlight their potential value for postgraduate education. Future multi-institutional and longitudinal studies are warranted to confirm the sustainability and generalizability of these outcomes across diverse contexts.

## Supplementary Information

Supplementary Material 1.

Supplementary Material 2.

Supplementary Material 3.

Supplementary Material 4.

Supplementary Material 5.

Supplementary Material 6.

Supplementary Material 7.

Supplementary Material 8.

## Data Availability

The author agree to disclose publicly for all available datasets presented in the main paper.

## Abbreviations

AI: Artificial Intelligence
CASP: Critical Appraisal Skills Programme
GRAMMS: Good Reporting of a Mixed Methods Study
LMS: Learning Management System
PAL: peer-assisted learning
