# Supplementary material for: Blended peer-led research curriculum with AI integration improves postgraduate students’ academic performance and satisfaction: a quasi-experimental mixed-methods study
Source: BMC Med Educ. 2026 Jan 19;26:260. doi: 10.1186/s12909-026-08576-2 (PMC12895863; doi:10.1186/s12909-026-08576-2)
Supplement: Supplementary file 1 — Supplementary Material 1. [file 12909_2026_8576_MOESM1_ESM.docx]

Good Reporting of A Mixed Methods Study (GRAMMS) checklist

| **Guideline** | **Where Addressed (Section & Page)** | **What Was Done in This Study** |
| --- | --- | --- |
| **Describe the justification for using a mixed methods approach to the research question** | Methods – Study Design, p. 7 | A mixed-methods approach was selected to evaluate not only whether the blended, peer-led, AI-integrated curriculum influenced academic performance and satisfaction (quantitative outcomes), but also to explore how and why students experienced these effects through qualitative insights. The qualitative component was intended to complement and elaborate quantitative findings by capturing learning processes, perceptions, and contextual factors not measurable through surveys or grades alone. |
| **Describe the design in terms of the purpose, priority, and sequence of methods** | Methods – Study Design, pp. 7–8 | A **convergent parallel mixed-methods design** was employed. Quantitative and qualitative data were collected concurrently during the same semester, with neither method prioritized over the other. Both strands addressed the same overarching research questions and were analyzed independently before being integrated at the interpretation stage. |
| **Describe each method in terms of sampling, data collection, and analysis** | Methods – Setting and Participants; Assessment Instruments; Data Analysis, pp. 7–10 | **Quantitative:** All enrolled postgraduate students in two course sections were included (intervention n=26; control n=26). Data included critique scores, final grades, and post-course satisfaction surveys. Analyses involved descriptive statistics, independent t-tests, and correlation analyses. **Qualitative:** Purposeful voluntary sampling was used for focus groups (one per cohort, 6–8 participants each). Data were collected via semi-structured focus groups and written reflections, transcribed verbatim, and analyzed using iterative thematic analysis following Braun and Clarke’s framework. |
| **Describe where integration has occurred, how it has occurred, and who has participated in it** | Methods – Study Design & Integration of Mixed Methods Findings, pp. 7–8, 13 | Integration occurred at the **interpretation stage**. Quantitative and qualitative findings were compared and contrasted to identify convergence and complementarity. Integration was achieved through narrative synthesis in the Results and Discussion sections, where qualitative themes were used to explain quantitative patterns (e.g., satisfaction, engagement). Integration was conducted by the single investigator responsible for both analyses. |
| **Describe any limitation of one method associated with the presence of the other method** | Discussion – Strengths and Limitations, pp. 22–24 | The qualitative component was limited by the small number of focus groups and potential social desirability bias due to facilitation by the course director. Quantitative measures were limited by the non-randomized design and the use of an investigator-developed satisfaction survey without quantitative content validity indices. Awareness of parallel curricula may also have influenced subjective ratings, potentially affecting integration of affective outcomes. |
| **Describe any insights gained from mixing or integrating methods** | Discussion – Implications & Interpretation, pp. 15–17, 19–21 | Integration revealed that while quantitative performance differences were modest, qualitative data provided explanatory insight into why satisfaction differed between groups, highlighting engagement, structure, peer interaction, and perceived relevance. Qualitative findings also identified workload-related tensions and contextual factors not captured by quantitative measures, demonstrating the added value of mixed methods in understanding educational interventions. |

O'Cathain A, Murphy E, Nicholl J. The quality of mixed methods studies in health services research. J Health Serv Res Policy. 2008;13: 92-98.
