# Supplementary material for: Blended peer-led research curriculum with AI integration improves postgraduate students’ academic performance and satisfaction: a quasi-experimental mixed-methods study
Source: BMC Med Educ. 2026 Jan 19;26:260. doi: 10.1186/s12909-026-08576-2 (PMC12895863; doi:10.1186/s12909-026-08576-2)
Supplement: Supplementary file 2 — Supplementary Material 2. [file 12909_2026_8576_MOESM2_ESM.docx]

**Rubric for Weekly Article Critiques**

| **Criterion** | **Definition** | **Score Range**  0–2 |
| --- | --- | --- |
| **Understanding of Content** | How well the critique demonstrates comprehension of the selected article’s key ideas, methods, and implications. |  |
| **Application of Course Concepts** | How effectively the critique applies concepts and skills learned in the course (e.g., research design, logic of argument, interpretation of findings). |  |
| **Analytical Depth** | The degree to which the critique goes beyond summary to provide reasoning, insight, and critical thinking. |  |
| **Clarity & Organization** | Coherence of written presentation, logical flow of arguments, and clarity of expression. |  |
| **Insight & Contribution** | Originality of interpretation and contribution to understanding the article’s strengths/limitations. |  |
|  | **Total possible score:** |  |
