# Supplementary material for: Blended peer-led research curriculum with AI integration improves postgraduate students’ academic performance and satisfaction: a quasi-experimental mixed-methods study
Source: BMC Med Educ. 2026 Jan 19;26:260. doi: 10.1186/s12909-026-08576-2 (PMC12895863; doi:10.1186/s12909-026-08576-2)
Supplement: Supplementary file 3 — Supplementary Material 3. [file 12909_2026_8576_MOESM3_ESM.docx]

Educational Intervention for Experimental Group

1. Flipped & Flexible Learning: Students accessed more than 50 self-produced YouTube lectures (mean duration ≈ 4.35 minutes, range 3–8 minutes), covering core research topics (e.g., hypothesis formulation, study design, literature review, critical appraisal), created by the course director [13].
2. Gamified Group-Based Critique: Each week, rotating groups of 6–7 students conducted article critiques designed to promote active learning and peer engagement. Emphasis was placed on providing constructive peer feedback, demonstrating originality, and applying analytical depth in evaluating the selected articles. Gamification elements—including competitive scoring and performance dashboards—were incorporated to enhance motivation and engagement. For example, each student was required to produce a *new* critique after reviewing all previous peer comments on the same article, ensuring progressive refinement of ideas. Weekly article critiques were evaluated by the course instructors using a standardized scoring rubric (Supplementary File S2) designed to reflect key learning outcomes of the course. The rubric included five criteria — understanding of content, application of course concepts, analytical depth, clarity and organization, and insight — each scored on a 0–2 scale for a maximum of 10 points. The criteria were shared with students in advance to enhance transparency and shared understanding of expectations. The group with the highest aggregate rubric score each week was identified as the “top-performing group.”

To mitigate evaluator bias given the instructor’s dual role as facilitator and evaluator, we implemented predefined rubric descriptors, as recommended in the rubric literature (e.g., Cornell Center for Teaching Innovation best practices on clear and measurable criteria)

AI tools—specifically ChatGPT, for which students received prior training—were permitted to support critique development by helping students improve clarity, organization, and language precision in their written analyses. Use of AI was restricted to ChatGPT and was intended to enhance communication rather than replace independent critical appraisal.

1. In-Class Application: Weekly class sessions included group tasks to design studies. A randomly selected student from each group presented the design to the entire class for peer and instructor feedback. Each group had 5-6 students.
2. CASP Peer Appraisal Rounds: Individually, students applied the CASP checklist to an article of their choice. Critiques were posted on the Blackboard learning management system within each group’s designated folder, where students could view one another’s submissions. Critiques were then exchanged within peer groups of 5 to 6 students for structured discussion. Discussions were guided explicitly by the CASP checklist, with students comparing their assessments item by item, identifying points of agreement or discrepancy, and collectively justifying their judgments. AI assistance was allowed during the critique preparation. Students were trained to use ChatGPT to enhance the clarity and structure of their written critiques and to refine the language used to justify their appraisal decisions. Use of AI was limited to ChatGPT only, and students were instructed to apply it as a tool to support—not replace—their critical reasoning. Discussions were guided explicitly by the CASP checklist, with students comparing their assessments item by item, identifying points of agreement or discrepancy, and collectively justifying their judgments.
3. Faculty-Led Discussion Rounds: Six thematic sessions were conducted, each centered on a specific study design (e.g., randomized controlled trials, cohort studies, systematic reviews/meta-analyses). Student peer groups did not use formal PowerPoint presentations; instead, they presented their CASP appraisals verbally. Each faculty member received the student critiques and the corresponding articles in advance, allowing them to review the materials thoroughly before the session. During the roundtable discussions, faculty rotated among the student groups, engaging each group in an in-depth verbal critique of their selected article, probing their reasoning, and offering expert guidance. This rotating roundtable format ensured that all students interacted directly with multiple faculty members and received rich, design-specific feedback on their critical appraisal work.
4. Online Expert Lectures: Live virtual sessions delivered by external experts on topics such as AI in research, grant writing, statistical decision-making, and mixed methods design.
5. Supplementary Skill Activities: Students developed comprehensive research proposals targeting real-world funding, completed a take-home research design examination, and obtained a certificate in research ethics. Students received coaching and support from peers as well as AI-assisted tools.
6. Final Proposal & Oral Presentation: Each student individually developed a comprehensive research proposal based on their own research interests and planned master’s thesis topic. Students refined their proposals using prior feedback from peers, AI tools, and instructors. They then defended their proposals orally before a faculty panel.
