# Supplementary material for: Blended peer-led research curriculum with AI integration improves postgraduate students’ academic performance and satisfaction: a quasi-experimental mixed-methods study
Source: BMC Med Educ. 2026 Jan 19;26:260. doi: 10.1186/s12909-026-08576-2 (PMC12895863; doi:10.1186/s12909-026-08576-2)
Supplement: Supplementary file 4 — Supplementary Material 4. [file 12909_2026_8576_MOESM4_ESM.pdf]

Research Methods DPH 806 | Course Evaluation 2024/2025

We'd really like to find out how you feel about our course last year!
Thank you, and again, your opinion is much appreciated!
The participation is voluntarily and it is going to be used for a research project.

* Indicates required question

We want to know which component of this course made a good impact in your learning

1. Class quiz *

Mark only one oval.

	1	2	3	4	5	
Not	◯	◯	◯	◯	◯	Very helpful

2. Having doctors to attend & give feedback to your presentation *

Mark only one oval.

	1	2	3	4	5	
Not	◯	◯	◯	◯	◯	Very helpful

3. Take home assignments *

Mark only one oval.

	1	2	3	4	5	
Not	◯	◯	◯	◯	◯	Very helpful

4. Group article critique discussion *

Mark only one oval.

	1	2	3	4	5	
Not	◯	◯	◯	◯	◯	Very helpful

5. Weekly article discussion *

Mark only one oval.

	1	2	3	4	5	
Not	◯	◯	◯	◯	◯	Very helpful

6. Class exam *

Mark only one oval.

	1	2	3	4	5	
Not	◯	◯	◯	◯	◯	Very helpful

7. In class activity *

Mark only one oval.

	1	2	3	4	5	
Not	◯	◯	◯	◯	◯	Very helpful

8. Online videos (if applicable) *

Mark only one oval.

	1	2	3	4	5	
Not	◯	◯	◯	◯	◯	Very helpful

9. Guest lectures (if applicable) *

Mark only one oval.

	1	2	3	4	5	
Not	◯	◯	◯	◯	◯	Very helpful

What do you think about the following:

10. Course objectives were clearly presented *

Mark only one oval.

	1	2	3	4	5	
Stro	◯	◯	◯	◯	◯	Strongly Agree

11. Course materials were well prepared *

Mark only one oval.

	1	2	3	4	5	
Stro	◯	◯	◯	◯	◯	Strongly Agree

12. The course significantly added to my existing knowledge *

Mark only one oval.

	1	2	3	4	5	
Stro	◯	◯	◯	◯	◯	Strongly Agree

13. Please comment on what were the most important skills you have gained because of this course *

14. On a scale of 0 to 10, how difficult was this course ? *

15. Do you believe there was an exceptional instructor in this course? If yes, who? and why? *

16. What did you like about this course? please be as specific as possible and add details. *

17. What did you dislike about this course? Please be as specific as possible and add details. *

What do you think about Dr. X

18. Knowledge of Subject Matter *

Mark only one oval.

	1	2	3	4	5	
Terr	◯	◯	◯	◯	◯	Outstanding

19. Value of Information Presented *

Mark only one oval.

	1	2	3	4	5	
Terr	◯	◯	◯	◯	◯	Outstanding

20. Length of Presentation: Did it start and end on time? *

Mark only one oval.

	1	2	3	4	5	
Terr	◯	◯	◯	◯	◯	Outstanding

21. Speaker Delivery: Was it engaging? Was the speaker loud enough? *

Mark only one oval.

	1	2	3	4	5	
Terr	◯	◯	◯	◯	◯	Outstanding

22. Content: Suitable detail for audience? Relevance to topic? Easy to follow? *

Mark only one oval.

	1	2	3	4	5	
Terr	◯	◯	◯	◯	◯	Outstanding

What do you think about Dr. Y

23. Knowledge of Subject Matter *

Mark only one oval.

	1	2	3	4	5	
Terr	◯	◯	◯	◯	◯	Outstanding

24. Value of Information Presented *

Mark only one oval.

	1	2	3	4	5	
Terr	◯	◯	◯	◯	◯	Outstanding

25. Length of Presentation: Did it start and end on time? *

Mark only one oval.

	1	2	3	4	5	
Terr	◯	◯	◯	◯	◯	Outstanding

26. Speaker Delivery: Was it engaging? Was the speaker loud enough? *

Mark only one oval.

	1	2	3	4	5	
Terr	◯	◯	◯	◯	◯	Outstanding

27. Content: Suitable detail for audience? Relevance to topic? Easy to follow? *

Mark only one oval.

	1	2	3	4	5	
Terr	◯	◯	◯	◯	◯	Outstanding

What do you think about Dr. Z

28. Knowledge of Subject Matter *

Mark only one oval.

	1	2	3	4	5	
Terr	◯	◯	◯	◯	◯	Outstanding

29. Value of Information Presented *

Mark only one oval.

	1	2	3	4	5	
Terr	◯	◯	◯	◯	◯	Outstanding

30. Length of Presentation: Did it start and end on time? *

Mark only one oval.

	1	2	3	4	5	
Terr	◯	◯	◯	◯	◯	Outstanding

31. Speaker Delivery: Was it engaging? Was the speaker loud enough? *

Mark only one oval.

	1	2	3	4	5	
Terr	◯	◯	◯	◯	◯	Outstanding

32. Content: Suitable detail for audience? Relevance to topic? Easy to follow? *

Mark only one oval.

	1	2	3	4	5	
Terr	◯	◯	◯	◯	◯	Outstanding

What do you think about Dr. W

33. Knowledge of Subject Matter *

Mark only one oval.

	1	2	3	4	5	
Terr	◯	◯	◯	◯	◯	Outstanding

34. Value of Information Presented *

Mark only one oval.

	1	2	3	4	5	
Terr	◯	◯	◯	◯	◯	Outstanding

35. Length of Presentation: Did it start and end on time? *

Mark only one oval.

	1	2	3	4	5	
Terr	◯	◯	◯	◯	◯	Outstanding

36. Speaker Delivery: Was it engaging? Was the speaker loud enough? *

Mark only one oval.

	1	2	3	4	5	
Terr	◯	◯	◯	◯	◯	Outstanding

37. Content: Suitable detail for audience? Relevance to topic? Easy to follow? *

Mark only one oval.

	1	2	3	4	5	
Terr	◯	◯	◯	◯	◯	Outstanding

38. Do you have recommendations for us to improve the course next year? *

____

____

____

____

____

This content is neither created nor endorsed by Google.

Google Forms
