# Supplementary material for: Blended peer-led research curriculum with AI integration improves postgraduate students’ academic performance and satisfaction: a quasi-experimental mixed-methods study
Source: BMC Med Educ. 2026 Jan 19;26:260. doi: 10.1186/s12909-026-08576-2 (PMC12895863; doi:10.1186/s12909-026-08576-2)
Supplement: Supplementary file 5 — Supplementary Material 5. [file 12909_2026_8576_MOESM5_ESM.docx]

**Rubric for Assignment 1**

|  | **Item** | **Criteria** | **Mark** |
| --- | --- | --- | --- |
| 1 | **Research Aim** | - Generalised overarching aim of the study | 1 |
| 2 | **Research Question** | - Sufficient statement that is enough to report a hypothesis (if applicable) - Clear, understandable, complete sentence where you can tell if it's descriptive or analytical (Relational, Causal, Comparison) - In a research question format | 3 |
| 3 | **Objectives** | - 1 primary objective and 1 secondary objectives - SMART objectives - Related to each other | 3 |
| 4 | **Hypothesis** | - Is it applicable? - If yes, the stated hypothesis should correctly reflect their main aim   - test cause-and-effect relationships   - look for differences among group means.   - check whether two variables are related without assuming cause-and-effect relationships | 1 |
| 5 | **References** | - Peer-reviewed journals - Consistent use of a referencing style | 2 |
|  | Total |  | 10 |
