# Supplementary material for: Blended peer-led research curriculum with AI integration improves postgraduate students’ academic performance and satisfaction: a quasi-experimental mixed-methods study
Source: BMC Med Educ. 2026 Jan 19;26:260. doi: 10.1186/s12909-026-08576-2 (PMC12895863; doi:10.1186/s12909-026-08576-2)
Supplement: Supplementary file 6 — Supplementary Material 6. [file 12909_2026_8576_MOESM6_ESM.doc]

**Assessment Rubric for Materials & Methods section**

|  | **Criterion/ Component** | **0** | **0.5** | **1** |
| --- | --- | --- | --- | --- |
|  | Study Design | Not mentioned  OR Inappropriate for research questions  OR Study design was not described. | Inappropriate given the research questions  OR Study design was not adequately described. | Appropriate and consistent with the research questions  +  Study design was adequately described |
|  | Sampling Methods | Not mentioned  OR Inappropriate  OR Sampling method was not described. | Sampling method was not adequately described. | Sampling method was appropriate and adequately described. |
|  | Study Population Characteristics | Not mentioned  OR Inappropriate  OR Characteristics and demographics of participants were not described. | Characteristics and demographics of  participants were not adequately  described. | Characteristics and demographics of  participants were adequately  described. |
|  | Study Variables | Not mentioned  OR Inappropriate  Study variables were not described AND not defined for measurement | Study variables were not adequately described  OR not well defined for measurement | Study variables were adequately described AND well defined for measurement |
|  | Data Collection Tools | Not mentioned  OR Inappropriate | Data collection was not adequately described. | Data collection was adequately described. |
|  | Procedures | Not mentioned  OR Inappropriate  Did not describe some steps in the execution of the research. | Did not adequately describe each step in the execution of the research. | Adequately described each step in  the execution of the research. |
|  | Statistical Analysis  ( if applicable) | Not mentioned  OR  Statistical analysis methods were not appropriate | Statistical analysis methods were not adequately described. | Statistical analysis methods were appropriate AND adequately described. |
|  | Ethical Considerations | Not mentioned | Confusing or incomplete | Mentioned & Complete |
|  | Writing style (Verb tense, voice, consistency of verb and object) | Paragraphs were of inappropriate sequence AND not concisely written | Paragraphs were of inappropriate sequence OR not concisely written | Paragraphs were of appropriate sequence AND concisely written |
| 10. | Mechanics (Grammar & Spelling) | There were more than 5 grammatical/ spelling mistakes. | There were up to 5 grammatical/ spelling mistakes. | There were 0-3 grammatical/ spelling mistakes. |
