# Supplementary material for: Blended peer-led research curriculum with AI integration improves postgraduate students’ academic performance and satisfaction: a quasi-experimental mixed-methods study
Source: BMC Med Educ. 2026 Jan 19;26:260. doi: 10.1186/s12909-026-08576-2 (PMC12895863; doi:10.1186/s12909-026-08576-2)
Supplement: Supplementary file 7 — Supplementary Material 7. [file 12909_2026_8576_MOESM7_ESM.docx]

**Supplementary File S4**

**Qualitative Codebook**

This codebook was developed through iterative open, axial, and thematic coding of four focus group transcripts. It was refined collaboratively by two independent coders and applied consistently across all data.

**Codebook Overview:**

- **Code**
- **Definition**
- **Inclusion Criteria**
- **Exclusion Criteria**
- **Example Quotes**

**1. Course Organization & Clarity**

**Definition:** Comments indicating that course structure, sequencing, or expectations were clear, logical, and easy to follow.
**Inclusion:** Mentions of syllabus clarity, well-organized content, explicit objectives.
**Exclusion:** Comments about instructor teaching style (see Code 2).
**Example:**

- “Everything was well organized and clear from the beginning.”

**2. Instructor Effectiveness & Support**

**Definition:** Perceptions of instructor approachability, clarity, engagement, or helpfulness.
**Inclusion:** Positive or negative remarks about teaching style, feedback quality.
**Exclusion:** Comments about peer interactions (see Code 5).
**Example:**

- “Prof X’s teaching style was excellent.”

**3. Active & Diverse Learning Methods**

**Definition:** Experiences with blended elements such as videos, in-class tasks, peer critique, and CASP appraisal.
**Inclusion:** Appreciation or criticism of variety and usefulness of methods.
**Exclusion:** Comments about workload or time constraints (see Code 8).
**Example:**

- “I liked the variety of exercises; each one taught me something new.”

**4. AI Tool Usability & Perceived Value**

**Definition:** Student reflections about using ChatGPT to refine critiques, language clarity, or reasoning.
**Inclusion:** Comments about usefulness, limitations, or impact of AI feedback.
**Exclusion:** General comments about feedback unrelated to AI (see Code 9).
**Example:**

- “AI helped me express my ideas more clearly.”

**5. Peer Collaboration & Group Dynamics**

**Definition:** Descriptions of teamwork, peer-to-peer critique, group discussion, and cooperative learning.
**Inclusion:** Positive or negative experiences interacting with peers.
**Exclusion:** Instructor feedback perceptions (see Code 2).
**Example:**

- “Working in groups was one of the most beneficial aspects.”

**6. Research Preparedness & Skill Development**

**Definition:** Statements indicating increased competency in designing studies, appraising evidence, or planning a thesis.
**Inclusion:** Mentions of feeling more confident or better prepared.
**Exclusion:** Comments focused on stress or difficulty (see Code 8).
**Example:**

- “It gave me a clue where to start planning my thesis.”

**7. Engagement & Motivation**

**Definition:** Descriptions of improved interest, enjoyment, or engagement through gamification, active learning, or feedback.
**Inclusion:** Enthusiasm due to specific course components.
**Exclusion:** Workload concerns (see Code 8).
**Example:**

- “The competitive scoring motivated me to improve.”

**8. Workload & Time Pressure**

**Definition:** Concerns about the number, difficulty, or pace of assignments, exams, or deadlines.
**Inclusion:** Descriptions of stress, being overwhelmed, or insufficient time.
**Exclusion:** Complaints about external speakers (see Code 10).
**Example:**

- “The number of assignments was too much.”

**9. Feedback Quality (Non-AI)**

**Definition:** Student perceptions of feedback from instructors or peers.
**Inclusion:** Helpful or unhelpful nature of critique, clarity of guidance.
**Exclusion:** Feedback generated by AI tools (see Code 4).
**Example:**

- “The feedback from my peers helped me understand my mistakes.”

**10. Course Logistics & Delivery Issues**

**Definition:** Problems related to scheduling, LMS (Blackboard), or session format.
**Inclusion:** Technical issues, unclear deadlines, scheduling conflicts.
**Exclusion:** Complaints about lack of relevance (see Code 11).
**Example:**

- “Blackboard was confusing.”

**11. Relevance & Alignment With Learner Needs**

**Definition:** Perceived mismatch between course level and student preparedness or specialty needs.
**Inclusion:** Statements about content being too advanced, too basic, or not specialty-aligned.
**Exclusion:** Workload complaints alone (see Code 8).
**Example:**

- “Some sessions started at a level beyond beginners.”
