# Supplementary material for: Blended peer-led research curriculum with AI integration improves postgraduate students’ academic performance and satisfaction: a quasi-experimental mixed-methods study
Source: BMC Med Educ. 2026 Jan 19;26:260. doi: 10.1186/s12909-026-08576-2 (PMC12895863; doi:10.1186/s12909-026-08576-2)
Supplement: Supplementary file 8 — Supplementary Material 8. [file 12909_2026_8576_MOESM8_ESM.pdf]

Informed Consent Form for Participation in Research

Invitation and Purpose

You are invited to participate in a research study evaluating two different instructional approaches for a postgraduate research-methods course. The aim is to compare a traditional lecture-based format with a blended, peer-led, and AI-augmented format to assess their effects on student satisfaction, learning experiences, and academic performance.
Participation in this research is voluntary.

What Participation Involves

If you agree to participate, you will be asked to:

- Complete the standard course requirements (assignments, critiques, proposal, oral defense) — identical for all students using the same rubrics and grading standards.
- If you are assigned to the intervention group, engage in peer-led critiques, group discussions, self-paced video learning, and optional use of an AI feedback tool.
- If you are assigned to the control group, participate in the traditional lecture-based curriculum.
- Complete a short post-course survey (5-point Likert scale) assessing your satisfaction, perceived learning gains, and, if relevant, your experience with the AI tool.
- Optionally (if you volunteer), take part in a focus-group discussion after course completion to reflect on your experiences; these discussions will be audio-recorded, transcribed, anonymized, and analyzed.

Your academic grade, evaluations, and standing in the course will **not** be affected by your decision to participate or not in the research components (survey/focus groups).
After course completion, students in the control group will be given access to all course materials used in the intervention group (videos, critique templates, etc.) for self-study, ensuring equitable opportunity.

Voluntary Participation & Right to Withdraw

- Participation is entirely voluntary.
- You may withdraw from the study at any time without penalty or any impact on your grades or academic standing.
- If you withdraw, any data you've provided will be kept only if you agree; otherwise, it will be removed.

Risks and Benefits

Risks:
There are no known physical risks. You may experience minor discomfort while discussing your learning experiences during focus groups. You may skip any question or withdraw at any time without consequences.
Benefits:
There may be no direct benefit to you personally. However, the study findings may help improve future research-methods curricula for dental students and better integrate active learning and AI tools in education.

Confidentiality and Data Protection

- All data collected (survey responses, focus-group transcripts, performance scores) will be anonymized — no names or student IDs will appear in any reports or publications.
- Electronic data will be stored in password-protected files; paper records, if any, will be kept in locked cabinets. Access will be limited to the research team.
- Audio recordings and transcripts of focus groups will be destroyed after analysis, or kept (anonymized) if required by institutional policy.

Use of AI Tools (for Intervention Group)

If enrolled in the intervention group, you may use an AI tool (like ChatGPT) to support critique writing, language refinement, or conceptual feedback. Use is optional, and final submissions must reflect your own critical thinking. Faculty will review all work for academic integrity and provide oversight.

Contact Information

If you have questions about the study, your rights, or wish to withdraw, you may contact:

Principal Investigator: Prof. Zuhair Natto, znatto@kau.edu.sa, Tel: +966xxxxxxxxx

---

Consent Statement

I have read and understood the information provided above. I have had the opportunity to ask questions and all of them have been answered to my satisfaction. I understand that participation is voluntary and that I may withdraw at any time without penalty. I consent to participate in this study.

Participant's Full Name: ____________________________

Signature: ____

Date: ____

Investigator's Name: _______________________________

Signature: ____

Date: ____
